# Recent Developments in Graphene/Polymer Nanocomposites for Application in Polymer Solar Cells

**DOI:** 10.3390/polym10020217

**Published:** 2018-02-22

**Authors:** Ana Maria Díez-Pascual, José Antonio Luceño Sánchez, Rafael Peña Capilla, Pilar García Díaz

**Affiliations:** 1 Department of Analytical Chemistry, Physical Chemistry and Chemical Engineering, Faculty of Biology, Environmental Sciences and Chemistry, Alcalá University, 28871 Madrid, Spain; jose.luceno@uah.es; 2 Department of Signal Theory and Communication, Polytechnic High School, Alcalá University, 28871 Madrid, Spain; rafa.pena@uah.es (R.P.C.); pilar.garcia@uah.es (P.G.D.)

**Keywords:** nanocomposite, conductive polymer, solar cell, graphene, graphene oxide, power-conversion efficiency, electrode, active layer, interfacial layer

## Abstract

Graphene (G) and its derivatives, graphene oxide (GO) and reduced graphene oxide (rGO) have enormous potential for energy applications owing to their 2D structure, large specific surface area, high electrical and thermal conductivity, optical transparency, and huge mechanical strength combined with inherent flexibility. The combination of G-based materials with polymers leads to new nanocomposites with enhanced structural and functional properties due to synergistic effects. This review briefly summarizes recent progress in the development of G/polymer nanocomposites for use in polymer solar cells (PSCs). These nanocomposites have been explored as transparent conducting electrodes (TCEs), active layers (ALs) and interfacial layers (IFLs) of PSCs. Photovoltaic parameters, such as the open-circuit voltage (*V*_oc_), short-circuit current density (*J*_sc_), fill factor (FF) and power-conversion efficiency (PCE) are compared for different device structures. Finally, future perspectives are discussed.

## 1. Introduction

Organic solar cells (OSCs) have received a lot of attention from both academia and industry in the last three decades since they exhibit important advantages compared to silicon-based devices such as their lightweight nature, flexibility, lower processing costs and lower environmental impact [1,2]. Furthermore, the active layers can be deposited through solution-based methods like spin-coating or printing, which allow massive device fabrication at low temperatures and, consequently, reduce device cost. OSCs are made of electron donor and electron acceptor materials. The material that absorbs the photons from the solar radiation is the donor, in which excited states (or excitons) are created and confined. An exciton is an electron-hole pair bound together by electrostatic interactions, which can be separated into free electron-hole pairs by effective electric fields. The material acquiring the electrons from the dissociated electron-hole pairs is the acceptor. 

According to the chemical structures of the active layer components, in particular the electron donor (p-type) semiconductors, OSCs can be further separated into two groups: polymer solar cells (PSCs) and small molecule solar cells. PSCs can also be transparent, which is of value for applications in windows, walls, flexible electronics, and so forth. Their main disadvantages are their low power-conversion efficiency (PCE) and photochemical degradation [3]. 

**Scheme 1:**
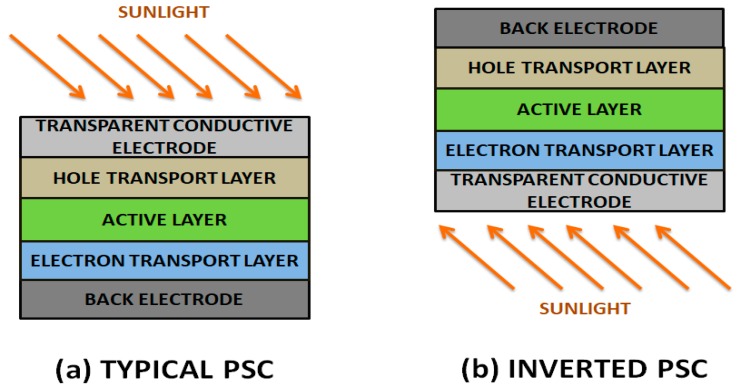
Schematic representation of a typical (**a**) and inverted (**b**) polymer solar cell (PSC).

PSCs are typically made of an indium tin oxide (ITO) conductive glass followed by a poly(3,4-ethylenedioxythiophene):poly(styrenesulfonate) (PEDOT:PSS) hole-transporting layer, an active layer (i.e., PBDTTT and PCBM), an electron transport layer (i.e., LiF) and, finally, a low work-function metal electrode like Al (Scheme 1). Conversely, in an inverted device, ITO with a buffer layer such as TiOx and ZnO is frequently used as the cathode electrode, and the anode is a high work-function metal like Ag or Au with a hole-transporting layer, i.e., PEDOT:PSS [4]. 

The nature and order of the layers as well as the nature of the metal electrode play a key role in the performance of the PSC device, hence on its PCE [5]. A bulk heterojunction (BHJ) structure is the most successful PSC architecture formed by blending an electron-rich conjugated polymer as donor and an electron-deficient fullerene as acceptor with a bicontinuous nanoscale interpenetrating network [6]. 

Three parameters are typically used to characterize a solar cell: (a) the open-circuit voltage (*V*_oc_), the maximum voltage available from a solar cell that occurs at zero current; (b) the short-circuit current density (*J*_sc_), the maximum current through the solar cell that occurs when the voltage is zero; (c) the fill factor (FF), which shows the extent to which current-voltage characteristics of the PSC are close to ideal; the optimal theoretical value of FF should be 1. In practice, FF values higher than 0.75 are regarded as very good. The overall performance or power-conversion efficiency (PCE) can be calculated based on these parameters and the incident solar power (*P*_in_) [7]. *V*_oc_ is principally restricted by the difference between the highest occupied molecular orbital (HOMO) of the polymer donor and the lowest free molecular orbital (LUMO) of the fullerene acceptor [8], and *J*_sc_ is determined by the product of the photoinduced charge-carrier density and the mobility within the organic semiconductors. 

**Figure 1:**
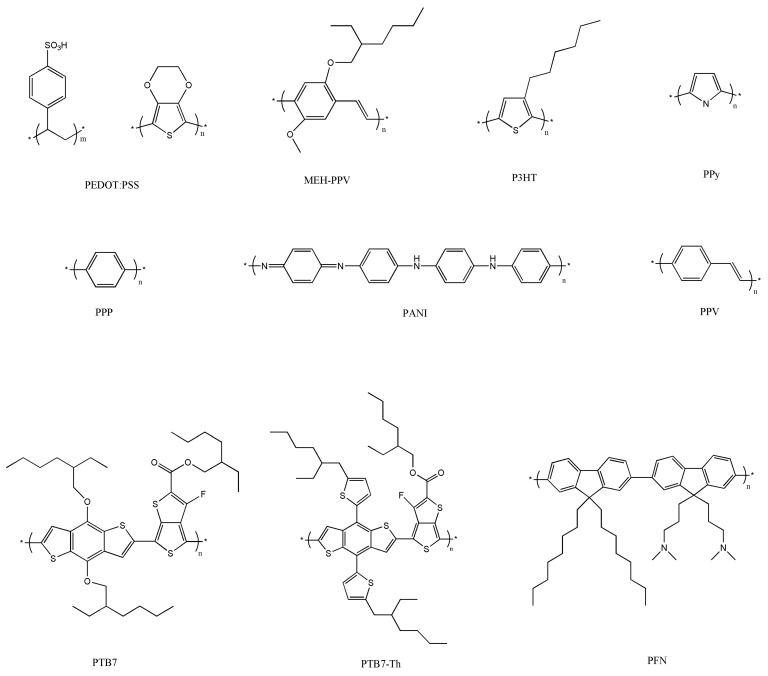
Chemical structure of conjugated polymers typically used in PSCs.

Over the last few years, considerable effort has been made to synthesize conjugated polymers and fullerene derivatives for use in BHJ cells [2,6]. They combine the electronic properties of traditional semiconductors and conductors with the ease of processing from solution, are lightweight, and have the mechanical flexibility of plastics. Figure 1 shows the chemical structure of some conjugated polymers widely employed in BHJ PSCs. The characteristic structure of alternating carbon–carbon double bonds is common to all of them, and it is responsible for their electronic properties, low-energy optical transitions, and high-electron affinities. As synthesized conjugated polymers (in a neutral state) are insulators, they become conductive by means of oxidation (*p*-doping) or reduction (*n*-doping). 

PEDOT is built from ethylenedioxythiophene (EDOT) monomers. It presents outstanding performance, including good electrochemical properties compared to those of other polythiophenes [9]. It is insoluble in many common solvents and unstable in its neutral state, since it oxidizes quickly in air. To enhance its processability, a polyelectrolyte solution, PSS is typically added, and this results in an aqueous dispersion of PEDOT:PSS, where PEDOT is its oxidized state. PSS acts as the counter ion and keeps the PEDOT chains dispersed in the aqueous medium. Nevertheless, PEDOT:PSS has several drawbacks like high acidity, hygroscopic character and inhomogeneous electrical properties, leading to low durability [10].

Poly(3-hexylthiophene) (P3HT) is one of the most widely used in photovoltaics due to its interesting electronic properties combined with its ability to self-assemble and to be easily dissolved in organic solvents. Poly(*p*-phenylene vinylene) (PPV) is hardly soluble. Attachment of side-groups to the conjugated backbone, as in poly[2-methoxy-5-(3′,7′-dimethyloctyloxy)-1,4-phenylene vinylene] (MDMO-PPV) or poly[2-methoxy-5-(2′-ethylhexyloxy)-p-phenylene vinylene] (MEH-PPV), significantly enhances the solubility of the polymer. However, PPV and its derivatives suffer from poor absorption and photodegradation [3]. The solubility of polypyrrole (PPy) is also restricted due to the cross-linking of the polymer chains. Neutral PPy is insoluble, though it can swell when exposed to some solvents. Once swelled, PPy can be doped either in acid or basic media and can be dissolved in a few solvents, such as chloroform, dimethyl sulfoxide (DMSO), *N*-Methyl-2-pyrrolidone (NMP), and tetrahydrofuran (THF). Poly(*p*-phenylene) (PPP) is only soluble in organic solvents, and is important since it is very stable (especially when undoped) and can be used as an electrode. Polyaniline (PANI) is especially attractive as it is relatively inexpensive, wasy to synthesise and environmentally stable. It has a wide range of electrical properties that can be easily controlled by changing its oxidation and protonation state and presents an acid/base doping response [11]. 

Initially, PSCs based on the BHJ structure used MEH-PPV as electron-donor and fullerene C60 derivatives as electron-acceptor, and were synthesized by spin-coating. However, the efficiency was relatively low (~7.7%) [12]. Over the last few years, the performance of these PSCs has been improved to a great extent, and can achieve PCEs > 9% for single-junction cells [13] and >11% for tandem solar cells [14]. This has been possible with the synthesis of new polymeric donor materials such as polythieno[3,4-*b*]thiophene-*co*-benzodithiophene] (PTB7). This low-bandgap semiconducting polymer has led to some of the highest reported efficiencies for BHJ cells due to its extended absorption into the near infra-red and lower HOMO level [13,15]. Furthermore, it exhibits high solubility in a wide range of solvents, which makes its processing very simple at room temperature, and it is also an excellent candidate for a variety of coating techniques including ink-jet printing, spraying and blade coating. In particular, a highly efficient PSC was obtained with an inverted device structure, where an alcohol-/water-soluble conjugated polymer, poly[(9,9-bis(3′-(*N*,*N*-dimethylamino)propyl)-2,7-fluorene)-alt-2,7-(9,9-dioctylfluorene)] (PFN) was used as the ITO surface modifier, and a blend of [6,6]-phenyl C71-butyric acid methyl ester (PC_71_BM) and PTB7 acted as the photoactive layer. Even an improved performance has been reported for poly[4,8-bis(5-(2-ethylhexyl)thiophen-2-yl)benzo[1,2-*b*:4,5-*b*′]dithiophene-*co*-3-fluorothieno[3,4-b]thiophene-2-carboxylate-2-6-diyl)], commonly known as PTB7-Th or PBDTTT-EFT. This displays lower HOMO/LUMO levels and increased efficiency compared to PTB7 and, more importantly, it is also far more stable. Thus, the PTB7-Th/PC_71_BM blend has been used as photoactive layer in single junction PSCs [16,17] because of its broadened photoresponse approaching 800 nm. Nonetheless, it has not been possible to further enhance the PCE of these cells since their manufacturing processes are difficult to scale up, and ion diffusion into the polymer layers along with their mechanical brittleness limit their applicability. Therefore, developing new materials with low cost and high stability that can be used as hole-transport layers or electron-transport layers in PSCs is of great interest.

In this regard, the present review summarizes recent progress in the development of graphene (G)/polymer nanocomposites for PSC applications. Due to its unique properties and versatility, G has emerged as a nanomaterial useful for several device parts including transparent conductive electrodes (TCEs), active layers (ALs) and the interfacial layers (IFLs). We will focus on the synthesis of G and its derivatives, graphene oxide (GO) and reduced graphene oxide (rGO), the preparation methods of G/polymer nanocomposites, and the photovoltaic properties of PSCs incorporating these nanocomposites. Although there are a number of reviews about PSCs [1,2,3,4,5,6], to the best of our knowledge literature reviews dealing with G-based materials for solar-cell applications are scarce [18,19], and none of them focuses on the nanocomposites. 

## 2. Graphene and its Derivatives: Synthesis and Properties

### 2.1. Synthesis and Properties of Graphene 

**Scheme 2:**
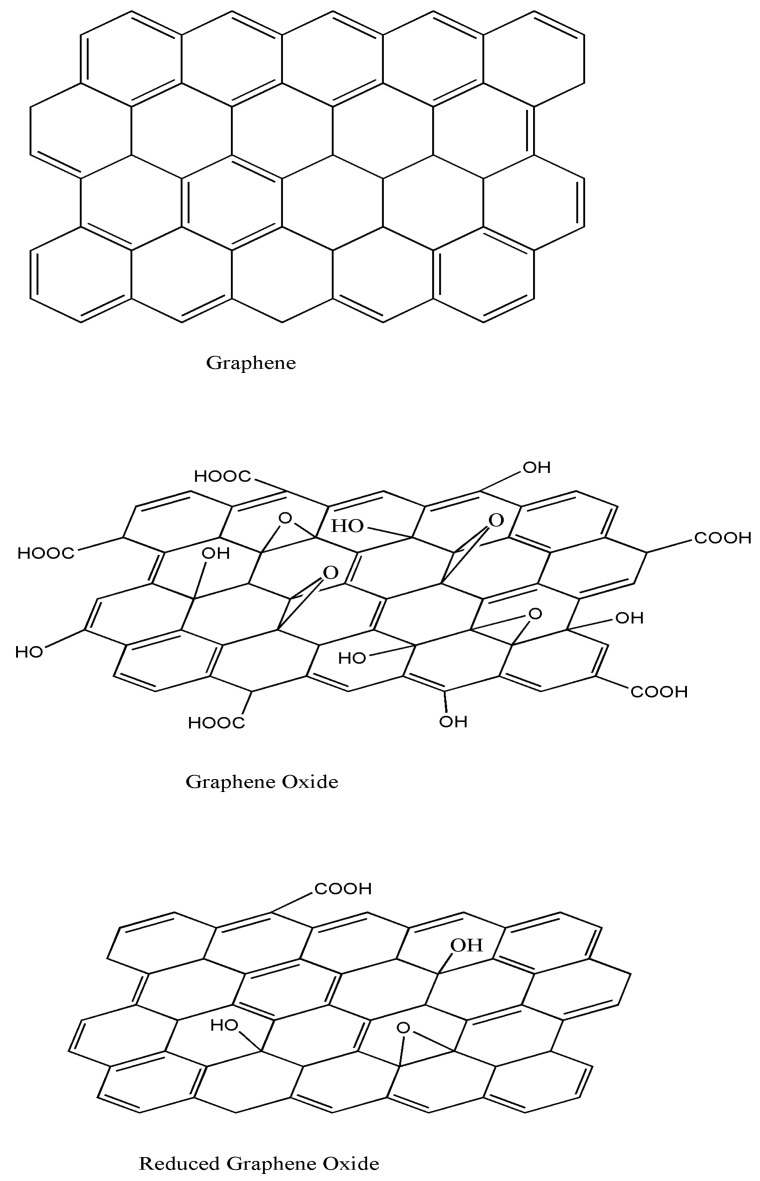
Schematic representation of the structure of graphene (G), graphene oxide (GO) and reduced graphene oxide (rGO).

As is well known, G consists of a flat, atomically thick single layer of sp^2^ carbon atoms forming a honeycomb structure (Scheme 2). Due to its 2D nature, its electronic band structure is characterized by a linear dependence of energy on momentum near the Fermi level [20]. G exhibits extraordinary electronic, thermal and mechanical properties [21], very high electron mobility (15,000 cm^2^/V s) and a very large surface area (2630 m^2^/g) [22]. It presents a thermal conductivity in the range of 3000–6000 W/m K [21], and an electrical conductivity of 9.6 × 10^7^ S/m. Moreover, G is one of the strongest materials on Earth, with an elastic modulus close to 1 TPa [23], an ultimate strength of 130 GPa, and a breaking strength of ~40 N/m [24]. Furthermore, the absorption of a G sheet is nearly constant, and equal to 2.3% [25]. The combination of these exceptional properties make G an excellent candidate for application in photovoltaic cells. Nonetheless, some issues need to be resolved. In particular, its sheet resistance is very high compared to those of materials currently used as electrodes such as ITO, hence it is important to enhance electronic transport without sacrificing its optical transparency and other properties. Besides, G sheets are flexible and chemically inert, which results in a double application: as an electrode and as a protective layer.

G can be synthesized by several techniques including exfoliation, epitaxial growth, chemical vapor deposition (CVD) and chemical and electrochemical methods. A brief description of these methods along with their feasibility for applications in solar energy devices are shown below.

Mechanical exfoliation was the first method developed to isolate G by peeling it off from graphite flakes using a Scotch tape. This method yields good quality G sheets; however, it is not suitable for mass production. 

Epitaxial growth is a substrate-based method, where G is grown on a single-crystal silicon carbide (SiC) by vacuum graphitization (thermal treatment of SiC at ~1300 °C under vacuum). The thickness of G layers can be controlled by adjusting the temperature and time, and the uniformity of the thickness improves with an annealing process [26]. This method is not practical for photovoltaic applications due to its relatively high cost and the strong adhesion of G layers to the substrate. 

CVD methods are the most widely used to fabricate G at a large scale, are the simplest, and are cost effective. G is grown directly on a transition metal substrate (Cu, Ni, Pt, Pd, Ru, Ir) by means of saturation of carbon upon exposure to a hydrocarbon gas (i.e., methane) at a high temperature [22]. When the substrate is cooled, the solubility of carbon on the substrate decreases and the carbon precipitates to form mono- to multilayer G sheets. The main disadvantages are the difficult control of the film thickness and the need for relatively expensive substrate materials. G grown on metals seems to be highly suitable for use in transparent electronics. 

Chemical-solution processes are inexpensive large-scale production methods to synthesize G for photovoltaic devices [27]. They provide a layered product that can be suitable for transparent electrodes or as dopant in other materials. The main shortcoming of these methods is the insolubility of G. However, the reaction with strong oxidizing agents, such as H_2_SO_4_, HNO_3_, or KMnO_4_, leads to the attachment of oxygen functional groups to both the basal plane and the edges of G sheets, resulting in the formation of GO (Scheme 2), which is soluble in various solvents, such as water, dimethylformamide (DMF), THF and chloroform [28]. Due to its dispersibility in common organic solvents, GO is highly suitable as a filler in polymeric nanocomposites. 

One of the least-used fabrication processes is electrochemical exfoliation, which relies on the penetration of graphite by ions from a solution forced by the applied potential. G obtained by this approach can be dispersed in organic solvents such as DMF, which enables the fabrication of thin films [29]. After reduction via treatment with HNO_3_ or under vacuum, layers with good transport and optical properties can be obtained, suitable for the fabrication of transparent electrodes.

### 2.2. Synthesis and Properties of Graphene Oxide

GO is an oxidized layer of G that contains epoxides, hydroxyls and carbonyls on the basal planes and carboxyls on the edges (Scheme 2). Consequently, some properties of GO differ from those of G: The sp^3^ carbon atoms in GO increase interlayer spacing, improving its ability to retain compounds. The attached groups and lattice defects modify the electronic structure of G and serve as strong scattering centers that affect electrical transport. Thus, it presents significantly lower electron mobility, and it is typically insulating, with a sheet resistance of about 10^12^ Ω/sq or higher. It presents aqueous processability, amphiphilicity, surface-functionalization capability, versatility, biocompatibility, and the ability to interact with biological cells and tissues [30]. More importantly, it is highly hydrophilic and can form stable aqueous colloids to facilitate the assembly of macroscopic structures, which is crucial for large-scale uses. Furthermore, it can be deposited on a substrate and later converted into a conductor, hence has great potential for the fabrication of solar cells. 

GO can be synthesized using four basic methods [26,30]: Staudenmaier, Hofmann, Brodie and Hummers. The most widely employed, the Hummers´ method, consists of the addition of KMnO_4_ to a solution of graphite, NaNO_3_ and H_2_SO_4_. Many variations of this method have been reported, with improvements constantly being explored to achieve better results and cheaper processes [26,30]. For instance, approaches without using NaNO_3_ eliminate the evolution of NO_2_/N_2_O_4_ toxic gasses and simplify the disposal of waste water because of the inexistence of Na^+^ and NO_3_^−^ ions [31]. 

GO can also be synthesized from graphite oxide by using sonication, stirring, or a combination of both. Sonication is a time-effective way of fully exfoliating graphite oxide, although it can seriously harm the graphene flakes, reducing their size from microns to nanometres, and even producing graphene platelets. Mechanical stirring is a less heavy-handed approach, albeit involving longer periods of time.

### 2.3. Synthesis and Properties of Reduced Graphene Oxide

An interesting property of GO is that it can be partly reduced to graphene-like sheets by eliminating the oxygen-containing groups with the recovery of a conjugated structure (Scheme 2). The reduced GO (rGO) sheets are regarded as functionalized G, chemically modified G or reduced G [32]. The aim is to attain graphene-like materials comparable to the pristine G obtained from direct mechanical exfoliation of graphite both in structure and properties. However, residual functional groups and defects considerably modify the structure of the carbon plane and, therefore, the properties of rGO differ from those of G. In particular, the electrical conductivity of rGO is typically in the range of 10–23 S/cm, much lower than that of G.

rGO can be prepared via the reduction of GO by thermal, chemical or electrical treatments. Thermal annealing consists in the reduction of GO only by heat treatment, via rapid heating (>2000 C/min) [33]. Exfoliation is caused by the abrupt expansion of CO or CO_2_ gases evolving into the spaces between graphene sheets during heating. This method cannot be used for GO films on substrates with a low melting point, such as glass and polymers, hence it is not suitable for solar cell applications. Thermal annealing can also be performed by microwave irradiation or via photo-reduction [34], using the energy emitted by a flash lamp or a laser. This process can lead to a much higher degree of reduction of GO, since the lamp/laser can provide higher energy than the thermal annealing, leading to rGO films with higher conductivity (i.e., 256 S/cm [35]), enabling the direct fabrication of electronic devices based on rGO films. 

Reduction by chemical reagents is based on their chemical reactions with GO, and can be carried out either at room temperature or applying moderate heat. The reagent more typically used is hydrazine monohydrate (N_2_H_4_·H_2_O), which is added to a GO aqueous dispersion, resulting in agglomerated rGO nanosheets due to the increase in hydrophobicity [36]. However, the toxicity of hydrazine and the possibility of incorporating N to the structure make it inappropriate for large-scale synthesis. Hence, other reducing agents have been proposed, such as NaBH_4_ [37], hydroiodic acid (HI), and urea. 

Electrochemical reduction of GO can be carried out in a usual electrochemical cell using an aqueous buffer solution at room temperature. The reduction does not require chemical agents, and it is induced by the electron exchange between GO and electrodes. This approach seems favorable for electrochemical applications. 

## 3. Preparation of Graphene/Polymer Nanocomposites

A variety of methods have been reported to prepare G/polymer nanocomposites including covalent and non-covalent approaches [24,38].

### 3.1. Non-Covalent Approaches

Non-covalent strategies include solution mixing, melt-blending and in situ polymerization. The solution technique requires both G material and polymer to be stably dispersed in a common solvent; it involves the dispersion of G in the appropriate solvent, the adsorption of the polymer on to delaminated G sheets in solution, and the elimination of the solvent, resulting in sandwich-like nanocomposites [38]. On the other hand, in the melt-blending process, G or its derivatives are blended into molten polymer matrixes under dramatic shearing. This process is not commonly used, particularly for energy applications. In the in situ polymerization, G is first swollen within the liquid monomer, the initiator is subsequently added and the polymerization begins either by heat or radiation. Nanocomposites with conductive polymers can also be produced via in situ electrochemical polymerization [39], which yields mechanically stable composite films that can be directly used as the electrodes of energy storage devices. 

### 3.2. Covalent Functionalization

In the covalent approach, the polymer chains are grafted on to the G sheets and wrap around them to prevent their aggregation. It can be carried out either via “grafting to” or “grafting from” approaches [40]. In the former, the functional groups of the polymer chains react with those of G or its derivatives to form chemical bonds. For instance, the carboxylic groups of GO can react with hydroxyl or amine groups of a polymer via esterification or amidation reactions [41]. The grafting from consists in the growth of the polymer chains from the surfaces of G sheets, typically via atom-transfer radical polymerization (ATRP). 

## 4. Graphene/Polymer Nanocomposites in Solar Cells

G-based materials have been used in different layers of PSCs including TCEs, ALs and IFLs. The literature on this subject is so extensive that only the most representative examples dealing with polymer/G nanocomposites are described. The most important results are summarized at the end of this section, in Table 1.

### 4.1. Graphene/Polymer Nanocomposites as Transparent Conductive Electrodes 

G and its derivatives have been used as TCEs to replace conventional ITO electrodes in PSCs. A lot of work has been carried out using flexible polymers, such as polyethylene terephthalate (PET) as substrate. In this regard, rGO films synthesized by thermal annealing were deposited on to PET, and the photovoltaic device was fabricated by spin coating the materials on to plasma treated rGO/PET to produce a hydrophilic surface [42]. A maximum PCE of 0.78%, *V*_oc_ of 0.56 V and *J*_SC_ of 4.39 mA/cm^2^ were attained when using rGO films with a thickness of 16 nm and a transmittance of 65%. Remarkably, the device obtained could withstand up to 1200 bending cycles without sacrificing device performance, whilst traditional cells incorporating ITO normally crack and degrade upon bending due to the brittle nature of ITO. Significantly improved performance (PCE of 3.05%) was obtained by depositing a rGO micromesh obtained via a laser-pattering technique on to PET, ascribed to the superior transparency (59%) and lower sheet resistance (565 Ω/sq) of the rGO micromesh compared to pristine rGO. More importantly, this PSC exhibited a comparable PCE with ITO-based devices (3.82%) and good bending stability [43]. The major drawback of these rGO-based electrodes is their high density of defects that limit device efficiency. 

**Figure 2:**
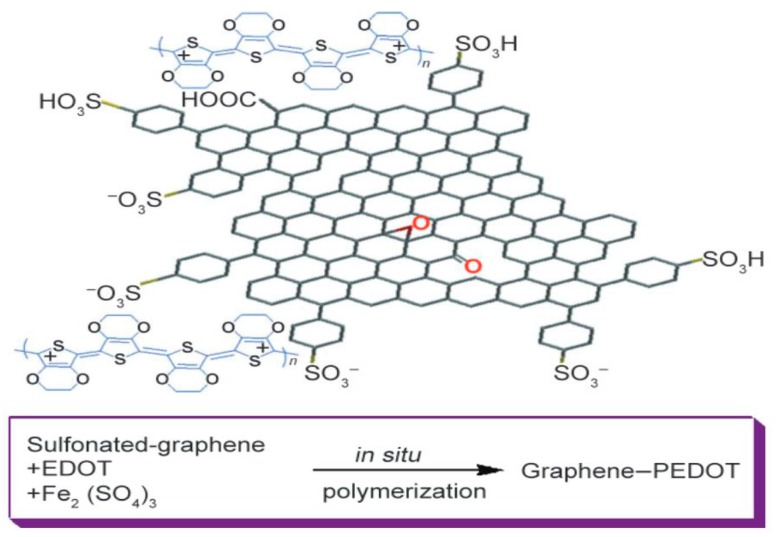
Schematic representation of sulfonated graphene (SG)/poly(3,4-ethylenedioxythiophene):poly(styrenesulfonate) (PEDOT) nanocomposite and its synthesis-reaction conditions. Reprinted from Ref. [44].

Xu et al. [44] reported the preparation of TCEs based on sulfonated graphene (SG)/PEDOT composites prepared by in situ polymerization (Figure 2). Firstly, SG was prepared from GO in four steps: (1) reduction of GO with NaBH_4_; (2) sulfonation with the aryl diazonium salt of sulfanilic acid; (3) post-reduction with N_2_H_4_; (4) functionalization with NaNO_3_, sulfanilic acid, and azoisobutyronitride (AIBN). Subsequently, SG was dispersed in water followed by the addition of Fe_2_(SO_4_)_3_ and the monomer EDOT. The mixture was stirred for 48 h at 50 °C, and then poured into methanol. The excess of EDOT and other impurities were removed through several washing cycles. The composites showed good processability both in water and organic solvents, superior transparency, high thermal conductivity, and thermal stability. A conductivity of 0.2 S/cm and transmittances higher than 80% in the wavelength range of 400–1800 nm were observed for films with thickness of a few nm. This conductivity is much higher than that of a commercial PEDOT:PSS product (10^−6^–10^−5^ S/cm). Moreover, when a poly(methyl methacrylate) (PMMA) sheet coated with this composite was bent inward, it still retained high electrical conductivity (0.18 S/cm).

A G-based composite electrode was prepared by spin coating a mixed solution of surfactant-functionalized G and PEDOT:PSS [45], with the modified G sheets uniformly distributed in the PEDOT:PSS matrix. The conductivity and transparency of this TCE were comparable to those of an ITO electrode. More importantly, it exhibited high stability (both mechanical and electrical), and it can be bent over 1000 cycles with only a 5% increase in its resistance. However, the presence of the surfactant stabilizer is undesirable from an application point of view. Jo et al. [46] synthesized an aqueous G suspension stabilized by PEDOT:PSS through the chemical reduction of GO in the presence of this polymer, without the need for surfactants. This approach involves strong π–π interactions between rGO sheets and the rigid backbone of PEDOT, and intermolecular electrostatic repulsions between negatively charged PSS bound to the RGO sheets, which impart colloidal stability to the resulting hybrid nanocomposite of rGO/PEDOT. The film exhibited a conductivity of 2.3 kΩ/sq with a transmittance of 80%. Moreover, its conductivity was approximately maintained after 100 bending cycles. 

Lima et al. [47] used a GO/PEDOT:PSS nanocomposite as TCE in a cell with structure PET/GO:PEDOT:PSS/F8T2/C_60_/Al, and a PCE of 1.10% was attained. The nanocomposite was fabricated without the need for any surfactant or dopant, and also played the role of hole-transport material in the device. By changing the GO/PEDOT:PSS ratio, the optical transmittance and sheet resistance of the nanocomposite films was tailored. Furthermore, the films showed good flexibility, without any conductivity reduction after 1000 bending cycles, and the procedure was compatible with large-scale fabrication processes. GO/PEDOT nanocomposites with potential applications as TCEs have also been recently synthesized by the Fenton’s reaction [48]. The synthesis was carried out using GO intercalated with FeCl_3_ and H_2_O_2_. Then, EDOT monomer was in situ polymerized on to the GO sheets. The resulting composite displayed improved electroactivity and optical properties.

Recently, a 4-layered CVD-doped G treated with PEDOT:PSS was applied as a cathode in inverted PSCs fabricated by spray coating [49]. The active layer was composed of poly[(5,6-difluoro -2,1,3-benzothiadiazol-4,7-diyl)-alt-(3,3‴-di(2-octyldodecyl)2,2′;5′,2″;5″,2‴-quaterthiophen-5,5‴-diyl)] (PffBT4T-2OD) as donor and phenyl-C71-butyric acid methyl ester (PC_70_BM) as acceptor. The resulting device showed a *V*_oc_ of 0.72 V, *J*_sc_ of 10.5 mA cm^−2^, FF of 0.37 and a PCE of 2.8%, respectively, not far from the corresponding values of an ITO-based cell. G-doped PEDOT:PSS nanocomposites have also been prepared by vibration-assisted ultrasonic spray coating [50,51], a single-step, fast, and scalable process. The resulting films showed a maximum electrical conductivity of 298 S·cm^−1^, about a 10-fold increase compared to pristine PEDOT:PSS films, with a transparency comparable to that of ITO-coated glasses. This extraordinary improvement was ascribed to the higher carrier mobility and carrier concentration, since G sheets bridge between the PEDOT:PSS rings via strong π–π interactions that act as high-mobility channels. Furthermore, the π–π interactions reduce the number of defect sites in PEDOT:PSS. More importantly, the energy level of G-doped PEDOT:PSS films could be tuned, and they exhibited better stability and superior mechanical properties, including wear resistance and hardness.

Sookhakian et al. [52] modified an ITO electrode with a G/ZnS/PPy ternary nanocomposite. prepared by layer-by-layer electrophoretic deposition. The resulting device showed a *V*_oc_ of 0.48 V, *J*_sc_ of 0.42 mA·cm^−2^, FF of 0.23 and a PCE of 0.92%, respectively, which were higher than those of devices with ZnS- or PPy-modified electrodes. The nanocomposite improved the photovoltaic efficiency because PPy acted as an exceptional sensitizer and hole acceptor, ZnS nanoparticles acted as bridges, whilst G acted as an outstanding conductive collector and transporter.

**Figure 3:**
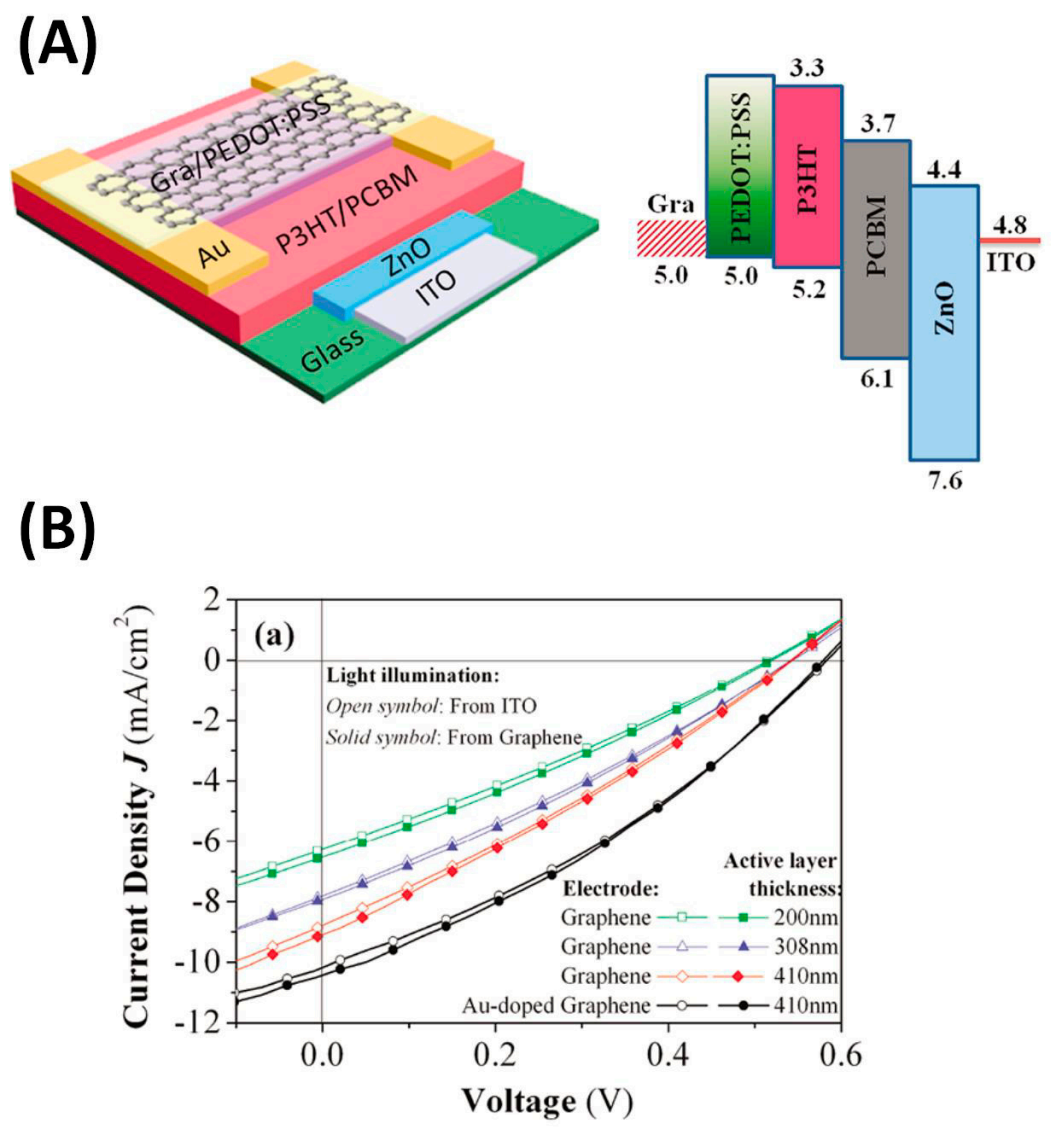
(**A**) Schematic representation and band structure of a PSC with the structure glass/indium tin oxide (ITO)/ZnO/P3HT:PCBM/Au/PEDOT:PSS/G; (**B**) J–V characteristics measured from two sides of the PSC with G top electrode and different active layer thicknesses. Reproduced with permission from Ref. [53].

Liu et al. [53] fabricated semitransparent PSCs based on P3HT: phenyl-C61-butyric acid methyl ester (PC_61_BM) with a CVD-grown single-layer G film doped with Au nanoparticles and PEDOT:PSS as the top electrode and ITO as the bottom electrode (Figure 3a). The use of the doped G electrode led to an increase in conductivity close to 400% compared to pristine G, and a maximum PCE of 2.7% was attained when illuminated from the G side (Figure 3b), which was attributed to the better transmittance of the G electrode. PSCs with higher efficiency are expected to be attained by optimizing the processing conditions and using single-layer G with superior quality.

The same authors [54] fabricated another flexible PSC on polyimide (PI) substrates with multilayer CVD G doped with PEDOT:PSS and Au nanoparticles as top TCE and P3HT:PC_61_BM as ALs. The device, with an structure of G/Au/PEDOT:PSS/P3HT:PCBM/ZnO/Ag/PI, showed a maximum PCE of ~3.2%, which diminished only by about 8% after 1000 bending cycles, indicating outstanding flexibility and stability. More importantly, it was found that air could not diffuse across the fine space among the G layers, thus providing an excellent packaging effect on flexible solar cells. Multilayer G can act as an environmental barrier and protect the PSCs from air contamination, which simplifies the device fabrication and reduces the associated costs. 

**Figure 4:**
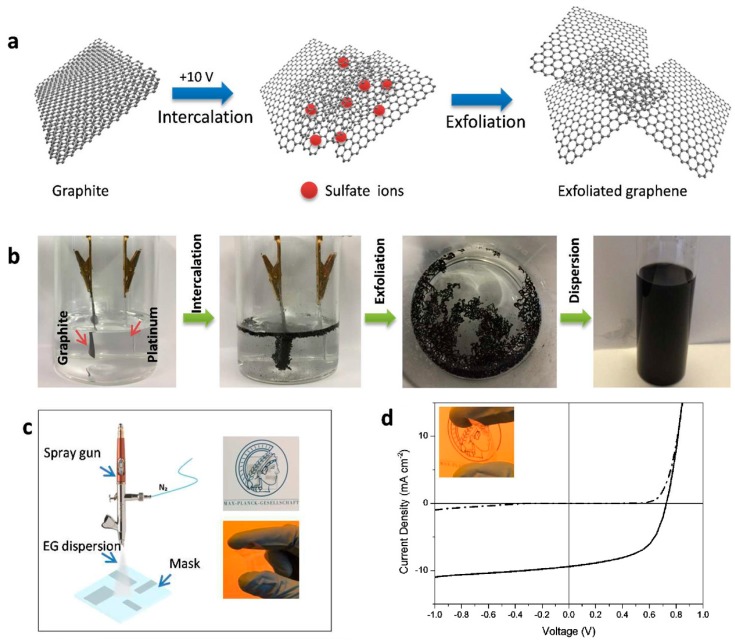
(**a**) Scheme of the electrochemical exfoliation of graphite; (**b**) optical images of the exfoliation process; (**c**) schematic representation of spray deposition of exfoliated graphene (EG) dispersion onto poly(ethylene 2,6-naphthalate) (PEN); (**d**) J–V characteristics of the cell under light (solid line) and dark conditions (dashed line). Reprinted with permission from [55].

Recently, G was prepared via electrochemical exfoliation of graphite [55] using an approach that intercalated sulfate ions (Figure 4). The resulting exfoliated graphene (EG) films were spray deposited on to flexible poly(ethylene 2,6-naphthalate) (PEN), and the composite was applied as anode in PSCs. The device using PTB7:PC_71_BM as the active layer yielded a PCE of 4.23%, which was kept after 150 bending cycles. This solution processed G-based electrode had a transparency of 70% and a low sheet resistance of 0.52 kΩ/sq, and was mechanically tough, without a change in resistance at different bending angles.

An et al. [56] developed another device incorporating a PMMA/G composite as anode. The polymer was spin coated onto highly uniform G prepared by CVD, resulting in a bilayer composite that was further deposited onto a flexible PET substrate. The PMMA improved the adhesion of G onto the substrate, preventing both contamination and crack formation. Based on this novel process, the G sheet resistance was reduced by about 50% at the same transmittance, leading to a device with a configuration of: PET/PMMA-G/MoO_3_/PEDOT:PSS/Poly[*N*-9′-heptadecanyl-2,7-carbazole-alt-5,5- (4′,7′-di-2-thienyl-2′,1′,3′-benzothiadiazole)] (PCDTBT):PC_70_BM/Ca/Al that exhibited a *V*_oc_ of 0.83 V, *J*_sc_ of 8.88 mA cm^−2^, FF of 0.45 and a PCE of 3.3%, about 200% higher efficiency than the reference cell without G. 

**Figure 5:**
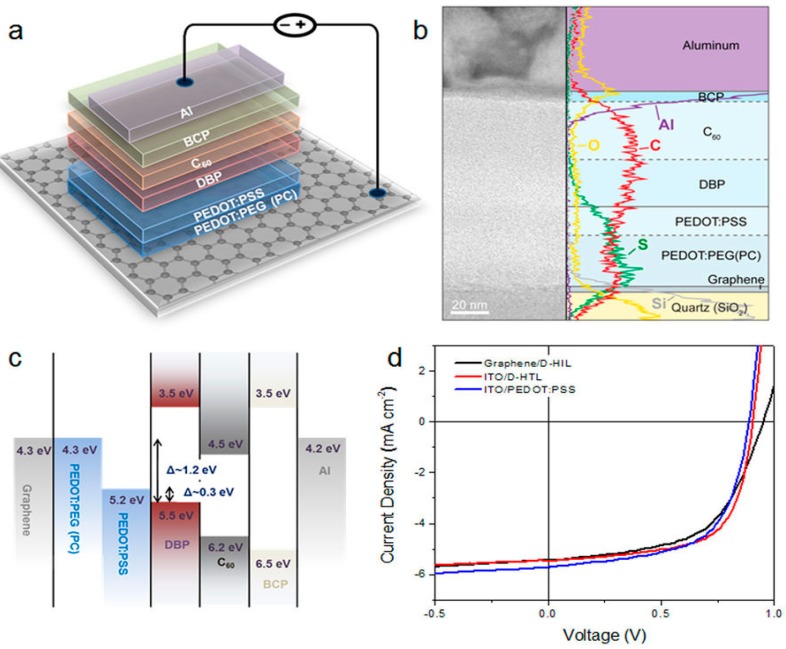
(**a**) Schematic representation of the PSC with G anode and structure G/PEDOT:PEG(PC)/PEDOT:PSS/DBP/C_60_/BCP/Al; (**b**) cross-sectional transmission electron microscope (TEM) image (left) of the device described in (**a**), with an energy-dispersive line scan on a diagram of the device cross-section (right); (**c**) flat-band energy level diagram of the PSC; (**d**) J–V characteristics of the G-based device compared to ITO reference cells (red and blue lines, respectively). Reproduced with permission from Ref. [57].

G has also been used as cathode and anode in PSCs. Park et al. [57] developed highly efficient flexible PSCs by modifying the G surface with a PEDOT-block-poly(ethylene glycol) (PEDOT:PEG) copolymer doped with perchlorate (PC) (Figure 5). The G films were fabricated via a low-pressure CVD (LPCVD) method, and comprised three monolayers. They had very good conductivity and transparency (resistivity of 300 Ω/sq and transmittance of 92%), and were applied as the anode in conventional cells and as the cathode in inverted devices [58], reaching PCEs of 6.1% and 7.1%, respectively. Bending tests demonstrated that these G-based devices were strong under mechanical deformation, without significant performance loss after 100 flexing cycles, and showed a maximum *V*_oc_ of 0.71 V, *J*_sc_ of 14.8 mA·cm^−2^ and a maximum FF of 57.6%. PSCs with LPCVD G as both anode and cathode have also been fabricated [59]. By using a PMMA-coated G as the bottom film and depositing the top G film onto flexible (PEN) substrates, a PCE of 3.7% was attained. 

### 4.2. Graphene/Polymer Nanocomposites as Active Layers

Initially, OSCs had an architecture similar to that of conventional solar cells: a single flat semiconductor heterojunction formed by a thin layer of active polymer (donor) and a film of acceptor, sandwiched between two electrodes. The field created in the donor–acceptor interface is responsible for exciton dissociation into free electron-hole pairs. The PCEs of OSCs based on this architecture is very low due to the reduced interface area. This means that only excitons formed very close to the interface (just a few nm) can dissociate into free-charge carriers. Therefore, flat hetorojunction OSCs must be thin, which leads to poor light absortion and low *J*_sc_.

Most OSCs developed later with significantly higher PCE where based on the BHJ architecture [6]. In this type of cell, an interpenetrating network with nanoscale phase separation in the active layer (consisting of a polymer donor and a fullerene-derivative acceptor) is formed. The PCE improvement in BHJ solar cells compared to bilayer solar cells is mainly due to the more efficient exciton dissociation enabled by the maximized heterojunction interface and increased charge-carrier collection due to the formation of the interpenetrating network. In this regard, the large specific surface area and 2D structure of G favor the formation of a bicontinuous interpenetrating network of donor and acceptor materials at the nanometer scale.

Liu and coworkers [60] reported the first use of solution-processable G functionalized with phenyl isocyanate as an acceptor and poly(3-octylthiophene) (P3OT) as a donor in PSCs. The device, with an ITO/ PEDOT:PSS/P3OT:G/LIF/Al structure, showed a maximum PCE of 1.4% for a G content of 5 wt % optimized via an annealing process (20 min at 160 °C). The same authors used such functionalized G in a higher concentration (10 wt %) as an acceptor and P3HT as donor in a similar PSC [61], with a maximum PCE of 1.1%, *V*_oc_ of 0.72 V, *J*_sc_ of 4.0 mA·cm^−2^ and FF of 0.38 after an annealing treatment at 160 °C for 10 min. The improvement in device performance was explained considering the expansion of the exciton dissociation area due to the faster electron transport through G. However, annealing at higher temperatures (i.e., 210 °C) resulted in a decrease in the PCE (0.57%). An analogous PSC using a P3OT/solution-processable G composite as the active layer was developed by Wang et al. [62]. The device showed a PCE of 1.14%, *V*_oc_ of 0.67 V, *J*_sc_ of 4.6 mA·cm^−^^2^, and FF of 0.37. Other authors prepared a P3HT/solution processable G/functionalized multiwalled carbon nanotubes (f-MWCNTs) nanocomposite, where P3HT acted as an electron donor, G as an electron acceptor and percolation path for the electrons, and the f-MWCNTs provided percolation paths of holes [63]. The resulting ITO/PEDOT:PSS/P3HT-f-MWCNTs-SPFGraphene/LiF/Al device showed a *J*_sc_ of 4.7 mA·cm^−^^2^, *V*_oc_ of 0.67 V, FF of 0.32, and a PCE of 1.05%. Nonetheless, the PCE of these cells with solution-processable G is significantly low, and could likely be improved by tuning the G content and processing conditions, since theoretical studies predict an efficiency above 12% for G-based PSCs.

**Figure 6:**
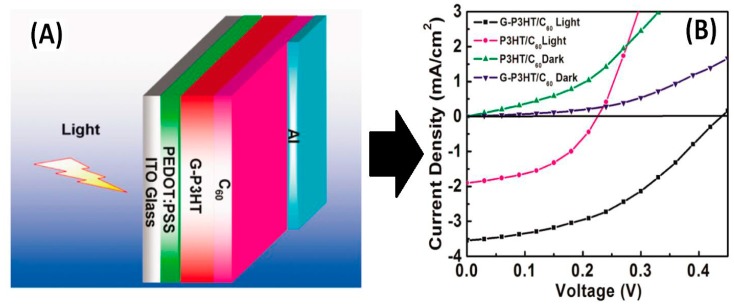
(**A**) Schematic representation of ITO/PEDOT:PSS/G-P3HT:C_60_/Al PSC; and (**B**) J–V characteristics using P3HT:C_60_ or G-P3HT:C_60_ as the active layer. Reproduced with permission from Ref. [64].

Yu et al. [64] grafted CH_2_OH-terminated regioregular P3HT on to the COOH groups of the GO surface via an esterification reaction. The resultant P3HT-grafted GO sheets were found to be soluble in a number of organic solvents, which is advantageous for solution processing. They used P3HT-*g*-GO/C_60_ as the active layer to build a photovoltaic device with the structure ITO/PEDOT:PSS/G-P3HT:C_60_/Al (Figure 6), showing a PCE of 0.61%, about 200% enhancement compared to the device based on P3HT/C_60_, since the chemical grafting of P3HT on to G can enhance electron delocalization and light absorption. The same group [65] grafted C_60_ on to the surface of rGO through a lithiation reaction, and the resulting hybrids were used as electron acceptors in the active layer to fabricate BHJ solar cells (device structure: ITO/PEDOT:PSS/C_60_-rGO:P3HT/Al). A maximum PCE of 1.22%, a *J*_sc_ of 4.45 mA cm^−^^2^ and a *V*_oc_ of 0.56 V were obtained, which are higher than those of many PSCs with non-fullerene electron acceptors, since the rGO–C_60_ hybrid can lead to a more effective electron transport pathway.

**Figure 7:**
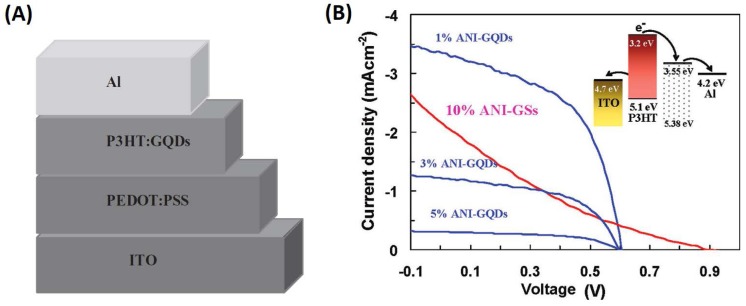
(**A**) Schematic representation; and (**B**) J–V curves of ITO/PEDOT:PSS/P3HT:GQDs/Al device based on aniline-modified GQDs with different GQDs content. Reproduced with permission from Ref. [66,67].

Converting 2D G into 0D quantum dots (GQDs) is a very effective approach to open the band gap of graphene. Furthermore, the GQDs are rich in oxygenated groups and soluble in aqueous and organic solvents, thus enabling further functionalization. GQDs with sizes of 3–5 nm, synthesized via an electrochemical approach, were used as electron-acceptors in a device with the structure of ITO/PEDOT:PSS/P3HT:GQDs/Al [66], leading to a *V*_oc_ of 0.67 V and a PCE of 1.28%. Aniline-modified GQDs were prepared via a hydrothermal method and used as acceptors in PSCs [67] with P3HT as donor (Figure 7), leading to a PCE of 1.14%. GQDs derived from double-walled carbon nanotubes have also been prepared via a solution-based method and incorporated into an active layer of P3HT:PCBM, leading to a PCE of 5.24% [68]. P3HT:PCBM:GQDs ternary composite represents a novel way to improve the efficiency of PSCs.

**Figure 8:**
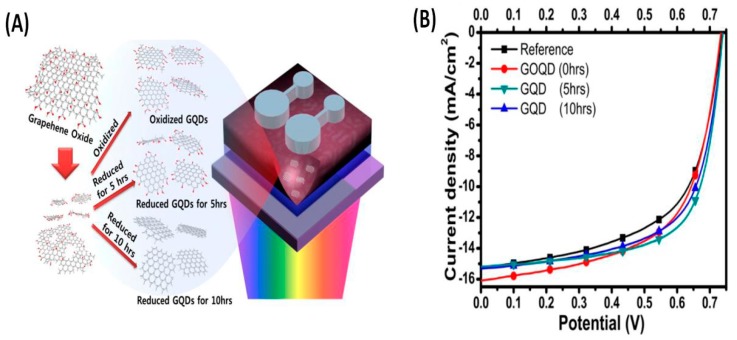
(**A**) Schematic representation of the synthesis of graphene oxide quantum dots (GOQDs) and reduced graphene oxide quantum dots (rGOQDs), where the edge functional groups are controlled by adjusting the thermal reduction time; (**B**) J–V curves of the PSCs with different types of GQDs. Reproduced with permission from Ref. [69].

To augment the optical absorptivity and charge-carrier extraction of PSCs, graphene oxide quantum dots (GOQDS) and reduced graphene oxide quantum dots (rGOQDs) have been blended with a PTB7:PC_71_BM active layer (Figure 8) leading to a device with structure ITO/PEDOT:PSS/GOQDs:PTB7:PC_71_BM/Al [69]. For rGOQDS, an increase in both *J*_sc_ and FF was observed due to the oxygenated functional groups on the surface of the QDs and their improved conductivity, leading to a maximum PCE of 7.6%. Another strategy to increase the band gap of G is doping with heteroatoms [70]. In this regard, PSCs have been developed by incorporating nitrogen-doped rGO in P3HT:PCBM ALs. The highest PCE obtained (4.5%) with this device (ITO/PEDOT:PSS/N-doped graphene:P3HT:PCBM/Al) was about 40% higher than that of a device without G. Overall, the performance of these PSCs with G-based materials is lower than that of conventional cells incorporating C_60_ and its derivatives [2,3,4]. Additional research should be carried out to control the structure and properties of G and optimize the device fabrication procedure to develop PSCs with higher PCE. 

Preliminary studies comparing the performance of G-based and carbon nanotube (CNT)-based PSCs revealed the superiority of those incorporating G. Thus, Lee et al. [71] compared the effect of maleimide-thiophene copolymer-functionalized GO (PTM21-GO) and carbon nanotubes (PTM21-CNTs) on P3HT/PCBM blends for use as active layers in PSCs. The performance was enhanced after incorporating both composites, and the highest PCE (1.70%) was obtained after the addition of 0.3 wt % PTM21-GO. The improved behaviour of the device with GO was attributed to the larger surface-contact area, higher charge-transporting capacity, along with the better dispersive nature after functionalization of the GO sheets compared to the CNTs. Furthermore, the amphiphilic character of GO makes its processability in aqueous solutions easier compared to CNTs that are hydrophobic and insoluble in most of the common solvents. 

### 4.3. Graphene/Polymer Nanocomposites as Interfacial Layers 

The IFLs, that is, the hole-transporting layer and electron-transporting layer between the active layer and the anode/or cathode, respectively, strongly condition the performance of PSCs. They are commonly used in order to improve the electrical contacts and enhance charge transport and collection. In addition, IFLs can improve light absorption and radiation distribution in the active layer and enhance performance stability. IFLs reduce charge-carrier recombination in electrodes by selectively allowing the desired carriers to pass through and preventing carriers from reaching the opposite electrodes. This is particularly important in BHJ PSCs, in which the donor and the acceptor semiconductors are randomly distributed in the active layer. Therefore, both semiconductors could be in contact with the anode and cathode, increasing the probability of recombination. IFLs can be designed in order to obtain a low contact resistance at the electrodes, avoiding this requirement to be met at the active layer, which can be designed without that restriction. IFLs also improve light distribution and absorption in PSCs by reducing the reflection of light at interfaces. 

G and its derivatives have been used as both types of layers, due to their benefits of superior energy-band structure that results in efficient charge transport and less corrosion for the ITO electrode [18]. A GO/PEDOT:PSS composite was used as the hole transport layer in a PCDTBT:PC_71_BM-based BHJ PSC, leading to a PCE of 4.28%, which is higher compared to devices with either GO or PEDOT:PSS as hole transport layers (PCEs of 2.77 and 3.57%, respectively). In addition, enhanced reproducibility and stability were observed. The improved performance was ascribed to the well-matched work function of GO and PEDOT:PSS that increased charge carriers’ mobility and reduced series resistance. Besides, GO could effectively block the electrons due to its large band-gap of ~3.6 eV, leading to an increased shunt resistance [72]. 

Chuang and Chen [73] developed a PEG-modified Au nanoparticles/GO nanocomposite as the hole-transport layer in a device with the structure: ITO/PEDOT:PSS(Au@PEG–GO)/PBDTTT-CT. PEG improved the solubility of the nanocomposite, so that it was well dispersed in water and several organic solvents. A relatively high PCE of 7.26% was attained. Besides, different spectral-enhancement regions were observed when the nanocomposite was placed at different locations, revealing the different dielectric environments nearby the nanoparticles, which could be useful for improving the broadband absorption of solar irradiation.

Jung et al. [74] synthesized a polyacrylonitrile-grafted rGO nanocomposite (PRGO) that acted as a hole-transport layer in PSCs based on PTB7-Th. The nanocomposite was fabricated via a covalent strategy based on in situ radiation-induced reduction and graft polymerization with acrylonitrile and styryl-functionalized GO. It displayed homogeneous thin-film morphology, good electrical conductivity (0.87 S/cm), high work function (4.87 eV), and exceptional weather stability. This combination of qualities makes it suitable as interfacial material in PSCs in order to improve photovoltaic efficiency and stability. The resulting device showed a PCE of 7.24%, FF of 0.64, J_sc_ of 14.78 mA/cm^2^, and *V*_oc_ of 0.76 V, comparable to those of a PEDOT:PSS-based one: PCE of 7.17%, FF of 0.66, *J*_sc_ of 13.91 mA/cm^2^, and *V*_oc_ of 0.78 V. More importantly, it exhibited superior durability.

**Figure 9:**
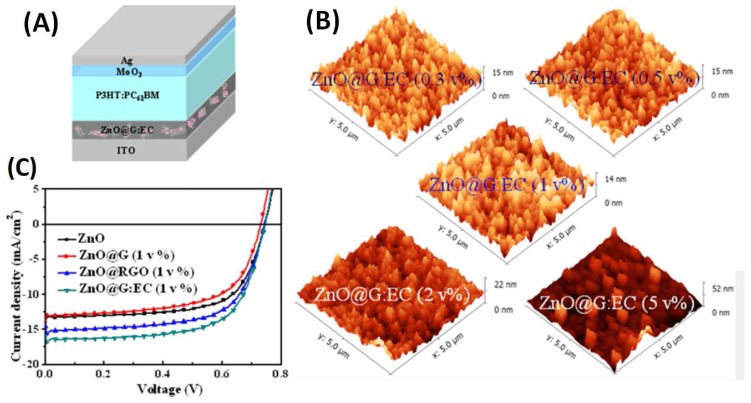
(**A**) Schematic representation of ITO/ZnO@G:EC/P3HT:PC_61_BM/MoO_3_/Ag device; (**B**) atomic force microscope (AFM) images of ZnO@G:EC nanocomposites with different G contents; (**C**) J–V curves of PSCs with the different nanocomposites. Reproduced with permission from Ref. [75].

Hu et al. [75] developed a new electron-transport layer based on ZnO nanocrystals dispersed in a G matrix with ethyl cellulose (EC) as a stabilizer. The ZnO@G:EC nanocomposites with different G contents exhibited a relatively smooth morphology (Figure 9), and retained the original structure of G with high conductivity. The device based on P3HT:PC_61_BM, with a structure of: ITO/ZnO@G:EC/P3HT:PC_61_BM/MoO_3_/Ag, showed a PCE of 3.9%, about 20% higher than that with bare ZnO nanocrystals. Replacing the active layer by PTB7:PC_71_BM, the PCE raised up to 8.4%. This simple approach can lead to highly conductive electron-transport layers for PSCs.

In another study, GO thin films were deposited by spin coating between a P3HT:PCBM active layer and ITO, causing a reduction in the recombination of electrons and holes as well as leakage currents [76]. The deposited GO acted as an effective hole-transport layer, and the best results were obtained for a GO film with a thickness of 2 nm. Thus, the device showed a PCE of 3.5%, a *V*_oc_ of 0.57 V, *J*_sc_ of 11.4 mA·cm^−2^ and a FF of 0.54. However, as the thickness of the films increased, the PCE decreased, due to the increased resistance of the hole-transport layer. In addition, spin coating such a thin GO film is extremely difficult. To solve these problems, nanocomposites with single-walled carbon nanotubes (SWCNTs) have been developed. Blending GO with SWCNTs significantly increased the conductivity of the nanocomposite; the device with a GO:SWCNT mass ratio of 1:0.2 exhibited a PCE of 4.1%, a *V*_oc_ of 0.6 V, *J*_sc_ of 10.8 mA·cm^−2^, and FF of 0.63, which are better than those of a PSC with raw GO as the hole-transporting layer. A nanocomposite of few-layer graphene nanosheets and PEDOT:PSS has also been used as a hole-transport layer, leading to an increase in the PCE from 3.1 to 3.7% compared to a conventional device with PEDOT:PSS [77]. In the case of butylamine-modified G/PEDOT:PSS nanocomposite, the PCE raised from 0.42 to 0.74%. Furthermore, when a GO/PEDOT:PSS nanocomposite was applied as the hole-transport layer, a PCE of 1.43 was attained, about 12% higher than the reference cell without GO, ascribed to the enhanced charge-carrier transport due to improved conductivity [78]. Overall, it seems that the addition of G derivatives as additives to PEDOT:PSS improves the performances of PSCs, although the reason for this enhancement is not fully understood yet. 

## 5. Conclusions and Outlook

G and its derivatives, GO and rGO have emerged as ideal materials for solar-cell applications owing to their 2D structure, outstanding electrical and thermal conductivity, high transparency, flexibility, superior mechanical strength, and very large specific surface area. In order to enhance their processability, chemical and electrochemical properties, and to expand their uses, G-based nanomaterials are frequently blended with polymers. In this regard, G/polymer nanocomposites seem to be useful in a range of device parts including transparent electrodes, active layers and interfacial layers. Nonetheless, while some improvements have already been attained when replacing conventional materials (i.e., ITO, PEDOT:PSS), several issues have to be addressed prior to the use of G-based nanocomposites as a key component in PSCs:

(a) The functionalization and ultrasonication processes applied to G prior to and during the fabrication of polymer nanocomposites leads to a strong reduction in electrical conductivity. Hence, the conductivity of chemically-derived G is considerably lower than that of ideal G. Consequently, G/polymer nanocomposites present poor electrical properties and do not satisfy the requirements for TCEs and counter electrodes in solar cells.
(b) Novel fabrication techniques to synthesize high-quality G thin films with tailored morphology and electronic properties are required, since the performance of G-based PSCs is directly related to the G characteristics: purity, quality, band-gap, structural morphology, etc. In the case of TCEs, solution-processable films with a good balance between high transparency and low sheet resistance are needed. More importantly, techniques that allow the synthesis of G at a large scale and at relatively low cost are highly desirable. Even though a lot of efforts have been made in this direction, the current methods are seriously limited by their low efficiencies, which should be addressed for commercial applications.
(c) The real specific surface area of G-based materials is much lower than the theoretical predictions owing to the strong agglomeration of the G sheets via π–π stacking interactions, and blending with polymers makes the problem even worse. Thus, new approaches to exfoliate the G sheets and keep a large specific surface area when blended with polymers are required. Although significant success has been attained via the addition of stabilizers such as surfactants, these frequently have detrimental effects on final device performance. Hence, novel synthetic routes are sought.
(d) Novel doping or functionalization strategies compatible with the fabrication process of PSCs have to be explored to attain high charge-carrier densities, better stability and tunable energy levels in G-based materials.
(e) The performance of G/polymer nanocomposites in PSCs is well below that reported for traditional materials. Thus, the composition and morphologies of the nanocomposites and the structure of the corresponding PSCs need further optimization. In this regard, developing new types of G-based materials such as GQDs is an interesting alternative. Owing to quantum confinement and the edge effect, the GQDs can exhibit better properties than pristine G, which could allow for the fabrication of PSCs with better PCE.
(f) The toxicity of nanostructured materials is well known [78,79] and the degradation caused by toxicity is of concern. G-based materials may cause cytotoxicity to humans, and this issue should be investigated in detail.

Overall, the research in this area is still in its infancy, and the mechanisms used to explain the observed effects are merely based on assumptions. Nonetheless, it is expected that after extensive research in the field and constant innovative efforts, PSCs incorporating G-based materials could offer a new outlook for the solar-energy industry. 

## Figures and Tables

**Table 1:** Characteristics of PSCs based on G/polymer nanocomposites.

Composite	Device Structure	*J*_sc_ (mA·cm^−2^)	*V*_oc_ (V)	FF	PCE (%)	REF
PET/rGO	PET/rGO/PEDOT:PSS/P3HT:PCBM/TiO_2_/Al	4.39	0.56	NA	0.78	42
GO:PEDOT:PSS	PET/GO:PEDOT:PSS/F8T2/C60/Al	2.75	0.71	0.52	1.10	47
Au/G/PEDOT:PSS	ITO/ZnO/P3HT:PCBM/PEDOT: PSS/Au/G	10.58	0.59	0.43	2.7	53
G/Au/PEDOT:PSS	G/Au/PEDOT:PSS/P3HT:PCBM/ZnO/Ag/PI	10.6	0.597	0.50	3.17	54
EG/PEDOT:PSS	EG/PEDOT:PSS/PTB7:PC71BM/Ba /Al	6.53	0.707	0.56	4.23	55
PMMA-G	PET/PMMA-G/MoO3/PEDOT:PSS /PCDTBT:PC_70_BM/Ca/Al	8.88	0.83	0.45	3.3	56
PEN/G	PEN/G/ZnO/PTB7:PC_71_BM/MoO_3_/Ag	14.9	0.71	0.58	6.2	58
P3OT:G	ITO/ PEDOT:PSS/P3OT:G/LiF/Al	4.2	0.92	0.37	1.4	60
P3HT:G	ITO/ PEDOT:PSS/P3HT:G/LiF/Al	4.0	0.72	0.38	1.1	61
P3OT:PCBM–G	ITO/PEDOT:PSS/P3OT:PCBM–G/LiF/Al	4.6	0.67	0.37	1.14	62
P3HT-f-MWCNTs-SPFG	ITO/PEDOT:PSS/P3HT-f-MWCNTs-SPFG/LiF/Al	4.7	0.67	0.32	1.05	63
P3HT-grafted GO	ITO/PEDOT:PSS/G-P3HT:C60/Al	3.5	0.43	0.41	0.61	64
C60-rGO:P3HT	ITO/PEDOT:PSS/C60-rGO:P3HT/Al	4.45	0.56	0.49	1.22	65
P3HT:GQDs	ITO/PEDOT:PSS/P3HT:GQDs/Al	6.33	0.67	0.30	1.28	66
P3HT/ANI-GQDs	ITO/PEDOT:PSS/P3HT/ANI-GQDs/Al	3.51	0.61	0.53	1.14	67
P3HT:PCBM:GQDs	ITO/PEDOT:PSS/P3HT:PCBM:GQDs/LiF/Al	26.46	0.60	0.33	5.24	68
GOQDs:PTB7: PC_71_BM	ITO/PEDOT:PSS/GOQDs:PTB7: PC_71_BM/Al	15.2	0.74	0.68	7.6	69
N-dopedG:P3HT: PCBM	ITO/PEDOT:PSS/N-doped G:P3HT:PCBM/Al	14.95	0.59	0.51	4.48	70
PTM21-GO	ITO/PEDOT:PSS/P3HT:PTM21-GO: PCBM/LiF/Al	8.11	0.59	0.30	1.43	71
GO/PEDOT:PSS	ITO/GO/PEDOT:PSS/PCDTBT: PC_71_BM/Al	10.44	0.82	0.50	4.28	72
Au@PEG–GO	ITO/PEDOT:PSS(Au@PEG–GO) /PBDTTT-CT	16.1	0.75	0.60	7.26	73
PRGO-PTB7-th	NA	14.78	0.76	0.64	7.24	74
ZnO@G:EC	ITO/ZnO@G:EC/P3HT:PC61BM/MoO3/Ag	7.73	0.63	0.73	3.9	75
GO/P3HT:PCBM	ITO/GO/P3HT:PCBM/Al	11.4	0.57	0.54	3.5	76
G:PEDOT:PSS	ITO/FLGs-PEDOT:PSS/PCBM/P3HT/Ca/Al	9.44	0.58	0.55	3.7	77

## References

[1] Brabec C.J., Sariciftci N.S., Hummelen J.C. (2001). Plastic solar cells. Adv. Funct. Mater..

[2] Cheng Y.-J., Yang S.-H., Hsu C.-S. (2009). Synthesis of conjugated polymers for organic solar cell applications. Chem. Rev..

[3] Jørgensen M., Norrman K., Krebs F.C. (2008). Stability/degradation of polymer solar cells. Sol. Energy Mater. Sol. Cells.

[4] Thompson B.C., Fréchet J.M. (2007). Polymer-Fullerene Composite Solar Cells. Angew. Chem. Int. Ed. Engl..

[5] Chen H.-Y., Hou J., Zhang S., Liang Y., Yang G., Yang Y., Yu L., Wu Y., Li G. (2009). Polymer solar cells with enhanced open-circuit voltage and efficiency. Nat. Photonics.

[6] Dennler G., Scharber M.C., Brabec C.J. (2009). Polymer-fullerene bulk-heterojuntion solar cells. Adv. Mater..

[7] Green M.A. (1981). Solar cell fill factors: General graph and empirical expressions. Solid State Electron..

[8] Luo J., Wu H., He C., Li A., Yang W., Cao Y. (2009). Enhanced open-circuit voltage in polymer solar cells. Appl. Phys. Lett..

[9] De Kok M.M., Buechel M., Vulto S.I.E., van de Weijer P., Meulenkamp E.A., de Winter S.H.P.M., Mank A.J.G., Vorstenbosch H.J.M., Weijtens C.H.L., van Elsbergen V. (2004). Modification of PEDOT:PSS as Hole Injection Layer in Polymer LEDs. Phys. Status Solidi A Appl. Res..

[10] Yun J.-M., Yeo J.-S., Kim J., Jeong H.-G., Kim D.-Y., Noh Y.-J., Kim S.-S., Ku B.-C., Na S.-I. (2011). Solution-processable reduced graphene oxide as a novel alternative to PEDOT:PSS hole transport layers for highly efficient and stable polymer solar cells. Adv. Mater..

[11] Taleghani H.G., Aleahmad M., Eisazadeh H. (2009). Preparation and Characterization of Polyaniline Nanoparticles Using Various Solutions. World Appl. Sci. J..

[12] Walker B., Kim C., Nguyen T.Q. (2011). Small molecule solution-processed bulk heterojunction Solar Cells. Chem. Mater..

[13] Das S., Keum J.K., Browning J.F., Gu G., Yang B., Dyck O., Chen W., Chen J., Ivanov I.N., Hong K. (2015). Correlating high power conversion efficiency of PTB7:PC71BM inverted organic solar cells with nanoscale structures. Nanoscale.

[14] You J., Dou L., Yoshimura K., Kato T., Ohya K., Moriarty T., Emery K., Chen C.-C., Gao J., Li G., Yang Y. (2013). A polymer tandem solar cell with 10.6% power conversion efficiency. Nat. Commun..

[15] Xue Z., Wang S., Yang J., Zhong Y., Qian M., Li C., Zhang Z., Xing G., Tao Y., Li Y., Huang W. (2018). Enhanced power-conversion efficiency in polymer solar cells using an inverted device structure. Npj Flex. Electron..

[16] Wan Q., Guo X., Wang Z., Li W., Guo B., Ma W., Zhang M., Li Y. (2016). 10.8% efficiency polymer solar cells based on PTB-Th and PC71BM via binary solvent additive treatment. Adv. Funct. Mater..

[17] Chen J.-D., Cui C., Li Y.-Q., Zhou L., Ou Q.-D., Li C., Li Y., Tang J.-X. (2014). Single junction polymer solar cells exceeding 10% power conversion efficiency. Adv. Mater..

[18] Lin X.-F., Zhang Z.-Y., Yuan Z.-K., Li J., Xiao X.-F., Hong W., Chen X.-D., Yu D.-S. (2016). Graphene-based materials for polymer solar cells. Chin. Chem. Lett..

[19] Sun Y., Zhang W., Chi H., Liu Y., Hou C.L., Fang D. (2015). Recent development of graphene materials applied in polymer solar cell. Renew. Sustain. Energy Rev..

[20] Wallace P.R. (1947). The band theory of graphite. Phys. Rev..

[21] Chang H., Wu H. (2013). Graphene-Based Nanomaterials: Synthesis, Properties, and Optical and Optoelectronic Applications. Adv. Funct. Mater..

[22] Weiss N.O., Zhou H., Liao L., Liu Y., Jiang S., Huang Y., Duan X. (2012). Graphene: An Emerging Electronic Material. Adv. Mater..

[23] Lee C., Wei X., Kysar J.W., Hone J. (2008). Measurement of the elastic properties and intrinsic strength of monolayer graphene. Science.

[24] Diez-Pascual A.M., Gomez-Fatou M.A., Ania F., Flores A. (2015). Nanoindentation in polymer nanocomposites. Prog. Mater. Sci..

[25] Nair R.R., Grigorenko A.N., Novoselov K.S., Booth T.J., Stauber T., Peres N.M.R., Geim A.K. (2008). Fine structure constant defines visual transparency of graphene. Science.

[26] Zheng Q., Kim J.K. (2015). Synthesis, Structure and Properties of Grpahene and Graphene Oxide. Graphene for Transparent Conductors. Synthesis, Properties and Applications.

[27] Wu J., Becerril H.A., Bao Z., Liu Z., Chen Y., Peumans P. (2008). Organic solar cells with solution processed graphene transparent electrodes. Appl. Phys. Lett..

[28] Díez-Pascual A.M., Díez-Vicente A.L. (2016). Poly(Propylene Fumarate)/Polyethylene Glycol-Modified Graphene Oxide Biocomposites for Tissue Engineering. ACS Appl. Mater. Interfaces.

[29] Su C.-Y., Lu A.-Y., Xu Y., Chen F.-R., Khlobystov A.N., Li L.-J. (2011). High-quality thin graphene films from fast electrochemical exfoliation. ACS Nano.

[30] Dreyer D.R., Park S., Bielawski C.W., Ruoff R.S. (2010). The Chemistry of Graphene Oxide. Chem. Soc. Rev..

[31] Chen J., Yao B., Li C., Shi G. (2013). An improved Hummers method for eco-friendly synthesis of graphene oxide. Carbon.

[32] Eda G., Chhowalla M. (2010). Chemically derived graphene oxide: towards large-area thin-film electronics and optoelectronics. Adv. Mater..

[33] Pei S., Chen H.-M. (2012). The reduction of graphene oxide. Carbon.

[34] Cote L.J., Cruz-Silva R., Huang J. (2009). Flash reduction and patterning of graphene oxide and its polymer composite. J. Am. Chem. Soc..

[35] Zhang Y., Guo L., Wei S., He Y., Xia H., Chen Q., Sun H.-B., Xiao F.-S. (2010). Direct imprinting of microcircuits on graphene oxides film by femtosecond laser reduction. Nano Today.

[36] Stankovich S., Dikin D.A., Dommett G.H., Kohlhaas K.M., Zimmey E.J., Stach E.A., Piner R.D., Nquyen S.T., Ruoff R.S. (2006). Graphene-based composite materials. Nature.

[37] Periasamy M., Thirumalaikumar M. (2000). Methods of enhancement of reactivity and selectivity of sodium borohydride for applications in organic chemistry. J. Organomet. Chem..

[38] Salavagione H.J., Díez-Pascual A.M., Lázaro E., Vera S., Gómez-Fatou M.A. (2014). Chemical sensors based on polymer composites with carbon nanotubes and graphene: The role of the polymer. J. Mater. Chem. A.

[39] Wang H.L., Hao Q.L., Xia X.F., Wang Z.J., Tian J., Zhu J.H., Tang C., Wang X. (2010). In Situ Fabrication of Nanoscale Graphene Oxide/Polyaniline Composite and its Electrochemical Properties. Adv. Mater. Res..

[40] Bai H., Li C., Shi G. (2011). Functional composite materials based on chemically converted graphene. Adv. Mater..

[41] Diez-Pascual A.M. (2017). Tissue Engineering Bionanocomposites based on Poly(propylene fumarate). Polymers.

[42] Yin Z., Sun S., Salim T., Wu S., Huang X., He Q., Lang Y.M., Zhan H. (2010). Organic Photovoltaic Devices Using Highly Flexible Reduced Graphene Oxide Films as Transparent Electrodes. ACS Nano.

[43] Konios D., Petridis C., Kakavelakis G., Sygletou M., Savva K., Stratakis E., Kymakis E. (2015). Reduced graphene oxide micromesh electrodes for large area, flexible, organic photovoltaic devices. Adv. Funct. Mater..

[44] Xu Y., Wang Y., Liang J., Huang Y., Ma Y., Wan X., Chen Y. (2009). A hybrid material of graphene and poly (3,4-ethyldioxythiophene) with high conductivity, flexibility, and transparency. Nano Res..

[45] Chang H., Wang G., Yang A., Tao X., Liu X. (2010). A Transparent, Flexible, Low-Temperature, and Solution-Processible Graphene Composite Electrode. Adv. Funct. Mater..

[46] Jo K., Lee T., Choi H.J., Park J.H., Lee D.J., Lee D.W., Kim B.-S. (2011). Stable aqueous dispersion of reduced graphene nanosheets via non-covalent functionalization with conducting polymers and application in transparent electrodes. Langmuir.

[47] Lima L.F., Matos C.F., Gonçalves L.C., Salvatierra R.V., Cava C.E., Zarbin A.J.G., Roman L.S. (2016). Water based, solution-processable, transparent and flexible graphene oxide composite as electrodes in organic solar cell application. J. Phys. D Appl. Phys..

[48] Carrasco-Valenzuela L., Zaragoza-Contreras E.A., Vega-Rios A. (2017). Synthesis of graphene oxide/poly(3,4-ethylenedioxythiophene) composites by Fenton’s reagent. Polymer.

[49] La Notte L., Bianco G.V., Palma A.L., Carlo A.D., Bruno G., Reale A. (2018). Sprayed organic photovoltaic cells and mini-modules based on chemical vapor deposited graphene as transparent conductive electrode. Carbon.

[50] Soltani-kordshuli F., Zabihi F., Eslamian M. (2016). Graphene-doped PEDOT:PSS nanocomposite thin films fabricated by conventional and substrate vibration-assisted spray coating (SVASC). JESTECH.

[51] Chen Q., Zabihi F., Eslamian M. (2016). Improved functionality of PEDOT:PSS thin films via graphene doping, fabricated by ultrasonic substrate vibration-assisted spray coating. Synth Met..

[52] Sookhakian M., Amin Y.M., Baradaran S., Tajabadi M.T., Golsheikh A.M., Basirun W.J. (2014). A layer-by-layer assembled graphene/zinc sulfide/polypyrrole thin-film electrode via electrophoretic deposition for solar cells. Thin Solid Films.

[53] Liu Z., Li J., Sun Z.-H., Tai G., Lau S.-P., Yan F. (2012). The Application of Highly Doped Single-Layer Graphene as the Top Electrodes of Semitransparent Organic Solar Cells. ACS Nano.

[54] Liu Z., Li J., Yan F. (2013). Package-Free Flexible Organic Solar Cells with Graphene top Electrodes. Adv. Mater..

[55] Ricciardulli A.G., Yang S., Feng X., Blom P.W.M. (2017). Solution-processable high-quality graphene for organic solar cells. ACS Appl. Mater. Interfaces.

[56] An C.J., Kim S.J., Choi H.O., Kim D.W., Jang S.W., Jin M.L., Park J.-M., Choi J.K., Jung H.-T. (2014). Ultraclean transfer of CVD-grown graphene and its application to flexible organic photovoltaic cells. J. Mater. Chem. A.

[57] Park H., Chang S., Smith M., Gradečak S., Kong J. (2013). Interface engineering of graphene for universal applications as both cathode and anode in organic photovoltaics. Sci. Rep..

[58] Park H., Chang S., Zhou X., Kong J., Palacios T., Gradečak S. (2014). Flexible graphene electrode-based organic photovoltaics with record-high efficiency. Nano Lett..

[59] Song Y., Chang S., Gradečak S., Kong J. (2016). Visibly transparent organic solar cells on flexible substrates with all-graphene electrodes. Adv. Energy Mater..

[60] Liu Z., Liu Q., Huang Y., Ma Y., Yin S., Zhang X., Sun W., Chen Y. (2008). Organic Photovoltaic Devices Based on a Novel Acceptor Material: Graphene. Adv. Mater..

[61] Liu Q., Liu Z., Zhang X., Yang L., Zhang N., Pan G., Yin S., Chen Y., Wei J. (2009). Polymer Photovoltaic Cells Based on Solution-Processable Graphene and P3HT. Adv. Funct. Mater..

[62] Wang H., He D., Wang Y., Liu Z., Wu H., Wang J. (2011). Organic Photovoltaic Devices Based on graphene as an electron-acceptor material and P3OT as a donor material. Phys. Status Solidi A.

[63] Liu Z., He D., Wang Y., Wu H., Wang J., Wang H. (2010). Improving photovoltaic properties by incorporating both SPFGraphene and functionalized multiwalled carbon nanotubes. Sol. Energy Mater. Sol. Cells.

[64] Yu D., Yang Y., Durstock M., Baek J.-B., Dai L. (2010). Soluble P3HT-Grafted Graphene for Efficient Bilayer–Heterojunction Photovoltaic Devices. ACS Nano.

[65] Yu D., Park K., Durstock M., Dai L. (2011). Fullerene-Grafted Graphene for Efficient Bulk Heterojunction Polymer Photovoltaic Devices. J. Phys. Chem. Lett..

[66] Li Y., Hu Y., Zhao Y., Shi G., Deng L., Hou Y., Qu L. (2011). An Electrochemical Avenue to Green-Luminescent Graphene Quantum Dots as Potential Electron-Acceptors for Photovoltaics. Adv. Mater..

[67] Gupta V., Chaudhary N., Srivastava R., Sharma G.D., Bhardwaj R., Chand S. (2011). Luminescent Graphene Quantum Dots for Organic Photovoltaic Devices. J. Am. Chem. Soc..

[68] Li F., Kou L., Chen W., Wu C., Guo T. (2013). Enhancing the short-circuit current and power conversion efficiency of polymer solar cells with graphene quantum dots derived from double-walled carbon nanotubes. NPG Asia Mater..

[69] Kim J.K., Park M.J., Kim S.J., Wang D.H., Cho S.P., Bae S., Park J.H., Hong B.H. (2013). Balancing Light Absorptivity and Carrier Conductivity of Graphene Quantum Dots for High-Efficiency Bulk Heterojunction Solar Cells. ACS Nano.

[70] Jun G.H., Jin S.H., Lee B., Kim B.H., Chae W.-S., Hong S.H., Jeon S. (2013). Enhanced conduction and charge-selectivity by N-doped graphene flakes in the active layer of bulk-heterojunction organic solar cells. Energy Environ. Sci..

[71] Lee R.-H., Huang J.-L., Chi C.-H. (2013). Conjugated polymer-functionalized graphite oxide sheets thin films for enhanced photovoltaic properties of polymer solar cells. J. Polym. Sci. B Polym. Phys..

[72] Rafique S., Abdullah S.M., Shahid M.M., Ansari M.O., Sulaiman K. (2016). Significantly improved photovoltaic performance in polymer bulk heterojuntion solar cells with graphene oxide/PEDOT:PSS double decked hole transport layer. Sci. Rep..

[73] Chuang M.-K., Chen F.-C. (2015). Synergistic Plasmonic Effects of Metal Nanoparticle–Decorated PEGylated Graphene Oxides in Polymer Solar Cells. ACS Appl. Mater. Interfaces.

[74] Jung C.-H., Noh Y.-J., Bae J.-H., Yu J.-H., Hwang I.-T., Shin J., Shin K., Lee J.-S., Choi J.-H., Na S.-I. (2017). Polyacrylonitrile-grafted reduced graphene oxide hybrid: An all-round and efficient hole-extraction material for organic and inorganic-organic hybrid photovoltaics. Nano Energy.

[75] Hu A., Wang Q., Chen L., Hu X., Zhang Y., Wu Y., Chen Y. (2015). In Situ Formation of ZnO in Graphene: A Facile Way To Produce a Smooth and Highly Conductive Electron Transport Layer for Polymer Solar Cells. ACS Appl. Mater. Interfaces.

[76] Li S.-S., Tu K.-H., Lin C.-C., Chen C.-W., Chhowalla M. (2010). Solution-processable graphene oxide as an efficient hole transport layer in polymer solar cells. ACS Nano.

[77] Nguyen D.D., Tai N.H., Chueh S.Y., Chen Y.J., Kuo W.S., Chou T.W., Hsu C.S., Chen L.J. (2011). Synthesis of ethanol-soluble few-layer graphene nanosheets for flexible and transparent conducting composite films. Nanotechnology.

[78] Hu X., Zhou Q. (2013). Health and Ecosystem Risks of Graphene. Chem. Rev..

[79] Chng E.L.K., Pumera M. (2013). The toxicity of graphene oxides: Dependence on the oxidative methods used. Chem.–Eur. J..

